# Mediterranean versus vegetarian diet for cardiovascular disease prevention (the CARDIVEG study): study protocol for a randomized controlled trial

**DOI:** 10.1186/s13063-016-1353-x

**Published:** 2016-05-04

**Authors:** Francesco Sofi, Monica Dinu, Giuditta Pagliai, Francesca Cesari, Rossella Marcucci, Alessandro Casini

**Affiliations:** Department of Experimental and Clinical Medicine, School of Human Health Sciences, University of Florence, Largo Brambilla 3, 50134 Florence, Italy; Unit of Clinical Nutrition, Careggi University Hospital, Florence, Italy; Don Carlo Gnocchi Foundation Italy, Onlus IRCCS, Florence, Italy; Unit of Atherothrombotic Diseases, Careggi University Hospital, Florence, Italy

**Keywords:** Diet, Prevention, Cardiovascular disease, Mediterranean, Vegetarian

## Abstract

**Background:**

Nutrition is able to alter the cardiovascular health of the general population. However, the optimal dietary strategy for cardiovascular disease prevention is still far from being defined. Mediterranean and vegetarian diets are those reporting the greatest grade of evidence in the literature, but no experimental studies comparing these two dietary patterns are available.

**Methods/design:**

This is an open randomized crossover clinical trial including healthy subjects with a low-to-medium cardiovascular risk profile, characterized by being overweight and by the presence of at least an additional metabolic risk factor (abdominal obesity, high total cholesterol, high LDL cholesterol, high triglycerides, impaired glucose fasting levels) but free from medications. A total of 100 subjects will be included and randomly assigned to two groups: Mediterranean calorie-restricted diet (*n* = 50) and vegetarian calorie-restricted diet (*n* = 50). The intervention phases will last 3 months each, and at the end of intervention phase I the groups will be crossed over. The two diets will be isocaloric and of three different sizes (1400 – 1600 – 1800 kcal/day), according to specific energy requirements. Adherence to the dietary intervention will be established through questionnaires and 24-h dietary recall. Anthropometric measurements, body composition, blood samples and stool samples will be obtained from each participant at the beginning and at the end of each intervention phase. The primary outcome measure will be change in weight from baseline. The secondary outcome measures will be variations of anthropometric and bioelectrical impedance variables as well as traditional and innovative cardiovascular biomarkers.

**Discussion:**

Despite all the data supporting the efficacy of Mediterranean and vegetarian diets on the prevention of cardiovascular diseases, no studies have directly compared these two dietary profiles. The trial will test whether there are statistically significant differences between these dietary profiles in reducing the cardiovascular risk burden for the general population.

**Trial registration:**

ClinicalTrials.gov NCT02641834

**Electronic supplementary material:**

The online version of this article (doi:10.1186/s13063-016-1353-x) contains supplementary material, which is available to authorized users.

## Background

Nutrition is able to substantially alter the health status of the general population [1]. In industrialized countries the most important association between diet and health is the relationship with cardiovascular disease, the leading cause of death and disability worldwide. Therefore, the ability to identify with certainty the relationship between diet and cardiovascular disease appears to be a key element in the implementation of specific primary prevention strategies [2]. Recent studies have focussed their interest on the impact of a whole dietary approach rather than on isolating single nutrients on the occurrence of cardiovascular disease; it is recognized that analyses of single nutrients ignore the important and complex interactions between components of a diet and, more importantly, ignore the fact that people do not eat isolated nutrients [3, 4]. However, an optimal dietary strategy for the prevention of cardiovascular disease has not been found [5].

Several models of diet have been imposed on public attention, but those that have received the most interest are the Mediterranean and vegetarian diets [6, 7]. These dietary patterns seem to exert protective effects on blood pressure, lipid profiles, cardiovascular diseases and metabolic parameters.

The term Mediterranean diet has been widely used to describe the traditional dietary habits of people in Crete, South Italy and other Mediterranean countries during the decade of the 1960s. This dietary pattern is characterized by plentiful plant-derived foods (fruits, vegetables, breads, other forms of cereals, beans, nuts and seeds), olive oil as the principal source of fat, moderate amounts of dairy products (mainly cheese and yogurt), low-to-moderate amounts of fish and meat and a moderate consumption, usually with meals. Since the results of the first data from the Seven Countries Study, several studies in different populations have established a beneficial role for the main components of the Mediterranean diet on the occurrence of cardiovascular and chronic degenerative diseases [8]. Recently, a meta-analysis conducted by our group has revealed, in a population of more than 2 million people, that a strict adherence to a Mediterranean dietary pattern is associated with a significant improvement in health status, as seen by a significant reduction in overall mortality (10 %) and incidence of and/or mortality from cardiovascular diseases (9 %) [9].

A vegetarian diet is a dietary profile characterized by abstention from consuming meat and meat products, poultry, seafood and flesh from any other animal. In the past few decades, some case-control and cohort prospective studies reported beneficial effects of this dietary profile on the occurrence of cardiovascular and neoplastic disease. Very recently, our group conducted a systematic review with a meta-analysis on more than 120,000 vegetarians, showing that adherence to a vegetarian dietary pattern helps to determine, among case-control studies, lower levels of the most important risk factor for chronic disease, along with a reduced risk of occurrence for ischemic heart disease (–25 %) when cohort prospective studies were taken into account [10].

Despite all these findings, however, to the best of our knowledge, no randomized controlled trial that compares, in the same group of patients, the efficacy of nutritional interventions based on Mediterranean and vegetarian dietary patterns is available in the literature. To date, it is unclear whether the supposed health benefits for vegetarians can be attributed to the absence of meat in the diet, to the increased consumption of particular food component(s), to the pattern of foods eaten within the vegetarian diet or to other healthy lifestyle components often associated with vegetarianism.

Hence, we aimed to design this randomized, open, crossover clinical trial that will test whether there is a difference between a vegetarian calorie-restricted diet and a Mediterranean calorie-restricted diet in reducing total weight and ameliorating the cardiovascular risk profile of a clinically healthy group of subjects.

## Methods/design

### Study design and setting

The randomized, open, crossover clinical trial will be conducted at the Unit of Clinical Nutrition of the Careggi University Hospital, Florence, Italy. A crossover design will be implemented to allow comparison of the vegetarian and Mediterranean diets within the same individual. Participants will act as their own controls in crossover studies, so individual differences will be controlled for, making the error variance smaller and subsequently reducing the sample size required to find a significant effect due to increased statistical power. This design will also be adopted in an effort to minimize attrition and to maximize participant interest and compliance by enabling each participant to experience both diet conditions. The study design follows the SPIRIT guidelines (see Additional files 1 and 2).

### Eligibility criteria

Eligible participants will be clinically healthy subjects (aged 18–75 years) with a low-to-medium cardiovascular risk profile (1–5 % according to the guidelines of the European Society of Cardiology) [11], determined by being overweight (body mass index >25.1 kg/m^2^) and by the concomitant presence of at least one of the following criteria:

- Waist circumference >88 cm (women) or >102 cm (men)
- Circulating levels of total cholesterol >190 mg/dL, not on drug treatment (measured no more than 3 months prior to the start of the study)
- Circulating levels of LDL cholesterol >115 mg/dL, not on drug treatment (measured no more than 3 months prior to the start of the study)
- Circulating levels of triglycerides >150 mg/dL, not on drug treatment (measured no more than 3 months prior to the start of the study)
- Circulating levels of fasting blood glucose >110 mg/dL but <126 mg/dL, not on drug treatment (measured no more than 3 months prior to the start of the study)

*Exclusion criteria* are the presence of a current serious illness or unstable condition that requires physician supervision of diet or physical activity (e.g. recent myocardial infarction, chronic liver disease, inflammatory bowel diseases); pregnancy or intention to become pregnant in the next 18 months; lactation; current or recent (past 6 months) participation in weight loss treatment program or use of weight loss medication; no regular intake of meat, fish or poultry for the past month.

### Interventions and participant timeline

This clinical randomized study of vegetarian and Mediterranean diets will use a crossover design with two intervention periods. After a 2-week run-in period, the eligible participants will be randomly assigned into two groups: either the group which will receive a vegetarian diet or the group which will receive a Mediterranean diet. Following the first intervention phase, the participants will be crossed over in order to obtain the second intervention phase. Each diet period will be 3 months long. The vegetarian diet will not contain meat, poultry or fish but will contain eggs and dairy, in addition to plant-based foods, such as fruits, vegetables, whole grains, legumes, nuts and seeds. The Mediterranean diet will contain all foods, including meat, poultry, fish, eggs, and dairy. However, red meat will be limited to once per week, poultry will be limited to ≤3 times per week and fish will be recommended two to three times per week. Both diets will derive approximately 25–30 % of energy from fat, 15–120 % from protein and the remainder from (primarily complex) carbohydrate. The vegetarian and Mediterranean diets will be calorie-restricted and isocaloric, supplying 1400 kcal/day for women and 1600 kcal/day for men with a weight <90 kg at baseline and 1600 kcal/day for women and 1800 kcal/day for men with a weight >90 kg at baseline. No meals or supplements will be provided. Participants will prepare their meals or eat at restaurants. For both diets, alcoholic beverages are limited to one per day for women and two per day for men. Participants will be asked not to alter their exercise habits during the study.

Standardized baseline assessment for both groups will include a questionnaire regarding demographic information, risk factors and co-morbidities. Moreover, at the baseline visit, participants will be educated about the aims and methods of the clinical trial. The subjects who agree to participate in the study, after signing the informed consent, will be included and randomly divided into two groups, each assigned to consume first either the vegetarian or Mediterranean diet. Each participant, before starting, needs to complete a 3-day dietary record (two weekdays and one weekend day). A dietician will analyse all the 3-day dietary records using a country-specific food nutrient database.

**Fig. 1:**
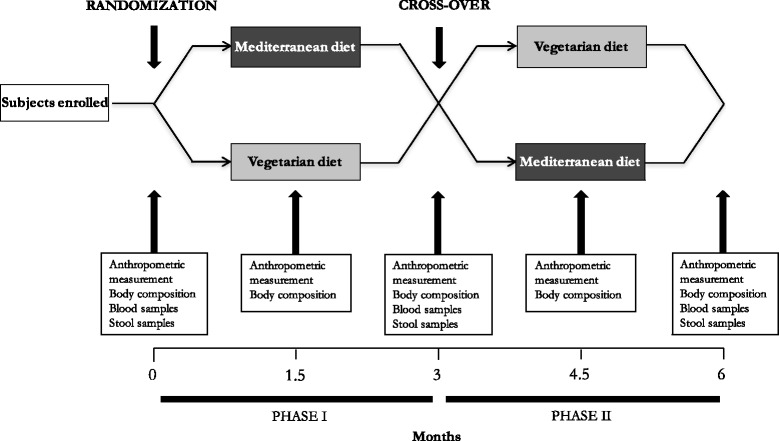
Organization of the intervention study

The study procedures are depicted in Fig. 1. There will be five clinical evaluations of the study population: one before the dietary intervention (*Time 0*), one 1.5 month after the start of the first dietary intervention (*Time 1*), one 3 months after the start of the first dietary intervention and at the time of intersection between the two groups (*Time 2*), one 4.5 months after the start of the study and 1.5 month from the intersection (*Time 3*), and the last one 6 months after the start of the study and 3 months from the intersection (*Time 4*). This design was chosen to ensure that there will be adequate time to achieve steady-state levels of lipid, glucometabolic, inflammatory and oxidative stress parameters.

Compliance with the intervention is critical to the success of this project and will be achieved using behaviour change strategies including self-monitoring and regular phone calls for dietary counselling. Furthermore, a dietician will provide the participants with a detailed 1-week menu plan for each dietary period, with all food ingredients expressed in weight and/or volume measures, and a handout containing details on their assigned diet, including food groups that can be included and ones that should be avoided. The vegetarian menu plan will also include recipes for preparing meals. Sessions for the two groups are similar in duration and content, except with regard to dietary details, and group leaders will be instructed to make no comment favouring one diet over the other or indicating their own dietary habits.

Participants may discontinue the intervention or withdraw from the study for the following reasons: (1) at the request of the participant or (2) if the investigator considers that a participant’s health will be compromised due to adverse events or concomitant illnesses that develop after entering the study.

### Outcomes

#### Primary outcome

The primary outcome is total weight change from baseline.

#### Secondary outcomes

The secondary outcomes are:

- Fat mass change from baseline
- Body mass index (BMI) change from baseline
- Lipid profile change from baseline (total cholesterol, LDL cholesterol, HDL cholesterol, triglycerides)
- Glycemic profile change from baseline (fasting glucose, insulin, HbA1c)
- Inflammatory parameters’ change from baseline [hs-CRP (high-sensitivity C-reactive protein), circulating levels of inflammatory cytokines: IL-1ra (interleukin-1ra), IL-1β (interleukin-1 beta), IL-2 (interleukin-2), IL-4 (interleukin-4), IL-6 (interleukin-6), IL-8 (interleukin-8), IL-10 (interleukin-10), IL-12 (interleukin-12), IL-17 (interleukin-17), IL-18 (interleukin-18), TNF-α (tumor necrosis factor alpha), IFN-γ (interferon gamma), VEGF (vascular endothelial growth factor), MCP-1 (monocyte chemoattractant protein-1), IP-10 (IFN-γ-inducible protein 10)]
- Oxidative stress markers’ change from baseline (total antioxidant profile, parameters of lipid peroxidation)

### Sample size

The sample size was determined on the basis of previous studies conducted to test the efficacy of a vegetarian diet on patients with type 2 diabetes mellitus [12]. To achieve an effect size between 1.25 and 2.1 for a power of 80 % at alpha < 0.05, for two groups, with a mean difference between groups from 2 to 3.5 kg and a standard deviation between 1.6 and 3.9, a sample size of at least 50 in each group of the study was required. It is projected that 100 subjects will be required in the final database and 10–25 % more must be randomized to achieve this number of completed subjects. These projections will be reviewed throughout the course of the study. Losses will be included in the intention-to-treat but not in the per-protocol analyses. The sample size calculation was performed using an online calculator software (available at http://www.powerandsamplesize.com).

### Recruitment and randomization

Male and female participants will be recruited by trial personnel using advertisements in local media, newspapers, social media, sheets and official websites. They will also recruit from a pre-existing database of participants and from friends or relatives of the hospital and university staff.

After approval and completion of the initial assessment, subjects will be formally entered into the study and randomized with a 1:1 randomization to study arms by a statistician, using a web-based online randomization procedure. Randomization will be stratified by age. The allocation concealment will be ensured using a centralized service, and it will not be possible for the investigators to know the allocation sequence in advance.

### Blinding

In this trial, blinding of participants and dieticians is not possible because of obvious differences between the intervention diets; however, outcome measures in the present study cannot be easily influenced by the observer. In addition, trial personnel who will enrol participants, outcome assessors and data analysts will be blinded to treatment allocation, and an employee outside of the research team will enter data into the computer in separate datasheets. On the other hand, making the trial open rather than blinded may improve recruitment.

### Data collection

Follow-up assessments and data collection will be undertaken at the Unit of Clinical Nutrition of the Careggi University Hospital, Florence, Italy, by trial personnel. All subjects will be examined between 6.30 and 9.30 a.m. after a 12-h fasting period.

#### Adherence and acceptability

In order to assess diet compliance, participants will be contacted at least twice during the study period. A dietician will make unannounced telephone calls to each participant to administer a 24-h diet recall. Adherence to the vegetarian diet will be measured as the absence of meat and fish from the dietary recalls. Participants in the Mediterranean group will be considered adherent if their food records report a great adherence (≥10 points on the 9-item literature-based Adherence Score Tool) to the Mediterranean diet.

At *Time 2* and *Time 4* participants will complete a modified version of the Food Acceptability Questionnaire asking participants to answer questions regarding the feasibility, the acceptability and the satisfaction/dissatisfaction with both diets. This questionnaire also asks respondents to indicate whether they have frequently experienced any of the following perceived benefits or adverse effects during the intervention period: changes in digestion and gastrointestinal function, increased or decreased energy, better or worse sleep than usual and self-perceived quality of life.

#### Anthropometric measurements and body composition

Weight and height will be measured using a stadiometer. Body mass index (BMI) will be calculated as weight (kg)/height (m)^2^. The body composition will be determined with a bioelectrical Body Composition Analyzer device (Tanita, model TBF-410) at baseline and follow-up visits.

#### Blood samples

Blood samples will be collected at the beginning and at the end of each intervention phase. Blood samples will be centrifuged at 3000 rpm for 15 minutes, aliquoted to yield serum, and then stored at –80 °C until analysis. The following biochemical measurements will be evaluated: complete blood count, lipid profile (total cholesterol, LDL cholesterol, HDL cholesterol, triglycerides), glucose profile (fasting glucose, insulin, HbA1c), liver function tests (aspartate aminotransferase, alanine transaminase, gamma-glutamyl transferase, alkaline phosphatase), renal function tests (serum creatinine, urea, uric acid), mineral profile (sodium, potassium, magnesium, calcium), iron metabolism (iron, ferritin), vitamin profile (vitamin B_12_, folic acid), pro-inflammatory and anti-inflammatory profile [hs-CRP (high-sensitivity C-reactive protein), circulating levels of inflammatory cytokines: IL-1ra (interleukin-1ra), IL-1β (interleukin-1 beta), IL-2 (interleukin-2), IL-4 (interleukin-4), IL-6 (interleukin-6), IL-8 (interleukin-8), IL-10 (interleukin-10), IL-12 (interleukin-12), IL-17 (interleukin-17), IL-18 (interleukin-18), TNF-α (tumor necrosis factor alpha), IFN-γ (interferon-gamma), VEGF (vascular endothelial growth factor), MCP-1 (monocyte chemoattractant protein-1), IP-10 (IFN-γ-inducible protein 10)], as well as antioxidant profile (total antioxidant profile, parameters of lipid peroxidation) and parameters related to vascular endothelial function such as circulating endothelial progenitor and circulating endothelial cell count.

#### Stool samples

Stool samples (four or five scoops totalling 4 g) will be collected before and after each intervention phase. Stool sample collection kits, including containers, will be provided for the participants. It is optional for the participants to provide the stool samples, so they can continue participating in the study even if they choose not to provide stool samples.

### Data management

Data will be collected in an electronic database. Identifiable data will not be recorded in the database or other documents, and participants will be identified by a unique trial ID only. Hard copies of data sheets linking the participant identification number to the person’s contact details will be kept securely in a locked filing cabinet in a locked office, accessible only to key research team members. Participant files and other source data (including copies of protocols, questionnaires, original reports of test results, correspondence, records of informed consent and other documents pertaining to the conduct of the study) will be kept for the maximum period of time permitted by the institution.

### Statistical analysis

Statistical analyses will be performed using SPSS software for Macintosh (SPSS Inc., Chicago, IL, USA). Outcomes will be analysed by intention-to-treat and on-treatment procedures. Histograms and box plots will be used to assess the distributional assumptions and to check for possible outliers. Log transformations will be applied, where appropriate, in order to render the outcome distributions closer to the normal. Bootstrap techniques will be used if this does not achieve reasonable normality to the extent that it may influence the properties of the regression analysis. Continuous variables that follow an approximately normal distribution will be summarized using the mean and standard deviations. Categorical variables will be presented in terms of frequencies and percentages. Before starting the data analysis, the level, pattern and likely causes of the missingness in the baseline variables and outcomes will be investigated by forming appropriate tables. This information will be used to determine whether the level and type of missing data has the potential to introduce bias into the analysis results or substantially reduce the precision of estimates for the proposed statistical methods.

All data will be treated as paired samples from a crossover study. The two interventions will be analysed by taking into account both periods in the two groups of subjects at different stages. The primary outcome, total weight change from baseline, will be analysed within each group using paired comparison *t* tests to test whether the changes from baseline to 3 months, from baseline to 6 months and from 3 to 6 months will be statistically significant. The absolute change (mean value at baseline subtracted from the mean value after intervention for each subject) will be estimated with independent *t* sample tests. One-way analysis of variance will be used for testing differences between changes in the vegetarian and Mediterranean intervention diet groups. A linear regression analysis (after checking regression assumptions), adjusting for age, gender, smoking habits and sedentary lifestyle, will be performed in order to compare the effect of the two different treatments. Data for the general linear model for repeated measurements will be reported as geometric means with their standard errors. A significant effect will indicate that the change from baseline to the vegetarian diet phase will be different from the change from baseline to the Mediterranean diet phase. Secondary outcome variables will be analysed similarly.

Multiple strategies will be employed to reduce attrition, including an accurate recruitment, a structured and time-limited protocol, the inclusion of a run-in period, the limitation of the burden and inconvenience of data collection on the participants and the development of a trusting and collaborative relationship between research units and participants. If participants do not attend a scheduled appointment, a maximum of three telephone calls will be made and one email sent prior to withdrawing the participant from the study. If a participant wishes to withdraw from the study intervention, the reason for withdrawal will be documented in the participant records for the subsequent analysis in the interpretation of the results.

Sensitivity analyses will be undertaken, based on the assumption that missing outcomes are the worst possible, or the best possible, in different randomization groups. If these show that conclusions may differ based on missing values, then supplementary multiple imputation for missing values will be undertaken. These analyses will account for results of any losses to follow-up insofar as they pertain to differences in measured variables (i.e. under the assumption of missing at random).

For questionnaire items related to acceptability, differences between responses during the vegetarian and Mediterranean diet phases will be tested using the Wilcoxon matched-pairs signed ranks test. The independent samples Mann-Whitney U test will be used to compare the diet groups. To test for differences in reported benefits and adverse effects of the diets, the chi-square test for independent samples will compare the two diet groups regarding frequency of changes in reported symptoms from baseline to final time points, and paired-comparison *t* tests will assess within-group changes over time. A *P* value < 0.05 will be considered statistically significant.

### Monitoring

Given the limited objectives and its short-term nature, this trial will be monitored by the protocol team and the local Institutional Review Board, without the use of a formal data monitoring committee. Data access will be restricted to trained staff with unique password-protected accounts. Adverse events such as unfavourable and unintended signs, abnormal laboratory findings, symptoms or diseases temporally associated with the intervention diet will be collected from the time of randomization until the final 6-month follow-up visit for each participant, whether or not considered related to the intervention study. All adverse events will be followed up until they are resolved.

### Ethics and dissemination

The study will be conducted in accordance with the Declaration of Helsinki and the Data Protection Act. Trial personnel will obtain informed consent from all participants prior to inclusion (see Additional file 3). All patients must agree to participate voluntarily and will be free to withdraw from the study at any time.

The study has received ethical approval by the Tuscany Regional Ethics Committee (REC) of the University Hospital of Careggi under the number SPE 15.054. REC approval included the trial protocol, information sheet and consent form, questionnaires, interviews, any other written information that will be provided to the participants and any advertisements that will be used during the study. The trial is registered at the clinical trial registration (ClinicalTrials.gov: NCT02641834, date of registration: 29 December 2015) in accordance with the International Committee of Medical Journal Editors (ICMJE) requirements.

Any amendments to the protocol and information provided to participants will be submitted to the REC for approval prior to implementation. Substantial amendments may only be implemented after written REC approval has been obtained, whereas non-substantial amendments can be implemented without written approval from the REC. The Chief Investigator will have to ensure that the participant’s privacy is maintained. Data and source documents will be stored in such a way that they can be accessed at a later date for the purposes of monitoring or inspection by the REC. At the end of the study, participants will be able to request a copy of the results of the study from the Chief Investigator.

The results from the trial will be submitted for publication in a peer-reviewed journal irrespective of the outcome. The final report will follow the CONSORT 2010 guidelines. Authorship of presentations and reports related to the study will be in the name of the collaborative group.

## Discussion

Because of the growing evidence in favour of the link between red and processed meat and cardiovascular disease risk [13], cancer [14], diabetes [15] and overall mortality [16], the interest in vegetarian diets has increased. Over the last few years, the number of subjects who began to adopt a plant-based dietary pattern has increased with respect to the past, when the population of vegetarians was limited to only a few and selected cohorts [17]. This increase has been mainly attributed to ethical and environmental motivations, as well as to health-related concerns [18].

There are several beneficial nutrients that are abundant in Mediterranean and vegetarian diets, such as monounsaturated fatty acids and high amounts of fiber and antioxidants; both diets also feature a low intake of total and saturated fats. However, to date, no studies are available that evaluate the effect of both diets in the same group of subjects at different time points. The comparison between vegetarian and Mediterranean dietary patterns in terms of cardiovascular risk prevention will be of paramount clinical significance for the general population and will help to improve understanding of the possible metabolic mechanisms underlying the health benefits associated with the adherence to either diet on ameliorating the cardiovascular risk profile of clinically healthy subjects.

## Trial status

At the time of manuscript submission, the enrolment of clinically healthy subjects is ongoing.

## Additional files

Additional file 1: SPIRIT Checklist. (DOC 120 kb) Additional file 2: SPIRIT time schedule. (PDF 35 kb) Additional file 3: Informed consent form. (DOC 220 kb)

## Abbreviations

BMI: body mass index
HbA1c: glycated haemoglobin
HDL: high-density lipoprotein
hs-CRP: high-sensitivity C-reactive protein
IL-1ra: interleukin-1 receptor antagonist
IFN-γ: interferon gamma
IL-1β: interleukin-1 beta
IL-2: interleukin-2
IL-4: interleukin-4
IL-6: interleukin-6
IL-8: interleukin-8
IL-10: interleukin-10
IL-12: interleukin-12
IL-17: interleukin-17
IL-18: interleukin-18
IP-10: IFN-γ-inducible protein 10
LDL: low-density lipoprotein
MCP-1: monocyte chemoattractant protein-1
REC: Regional Ethics Committee
TNF-α: tumor necrosis factor alpha
VEGF: vascular endothelial growth factor
