# Erratum to: ‘Mediterranean versus vegetarian diet for cardiovascular disease prevention (the CARDIVEG study): study protocol for a randomized controlled trial’

**DOI:** 10.1186/s13063-016-1395-0

**Published:** 2016-05-19

**Authors:** Francesco Sofi, Monica Dinu, Giuditta Pagliai, Francesca Cesari, Rossella Marcucci, Alessandro Casini

**Affiliations:** Department of Experimental and Clinical Medicine, School of Human Health Sciences, University of Florence, Florence, Italy; Unit of Clinical Nutrition, Careggi University Hospital, Florence, Italy; Don Carlo Gnocchi Foundation Italy, Onlus IRCCS, Florence, Italy; Unit of Atherothrombotic Diseases, Careggi University Hospital, Florence, Italy

Unfortunately, the original version of this article [1] contained an error. The presentation of Fig. 1 was incorrect. The problem is with the positioning of the squares where Mediterranean and vegetarian diet are written. In the figure, after the crossover the squares should be swapped so that the diet is swapped. The correct version of Fig. 1 is included here.

**Fig. 1:**
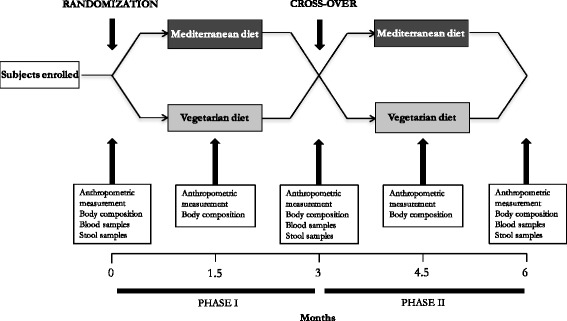
Organization of the intervention study
