# Supplementary material for: Mediterranean versus vegetarian diet for cardiovascular disease prevention (the CARDIVEG study): study protocol for a randomized controlled trial
Source: Trials. 2016 May 4;17:233. doi: 10.1186/s13063-016-1353-x (PMC4855805; doi:10.1186/s13063-016-1353-x)
Supplement: Additional file 2: — SPIRIT time schedule. (PDF 35 kb) [file 13063_2016_1353_MOESM2_ESM.pdf]

Figure. **Time schedule of enrolment, interventions, and assessments for participants**

	STUDY PERIOD					
	Enrollment	Allocation	Post-allocation			End of the study
**PROTOCOL ACTIVITY**	*day-14 to 1*	*day 0*	*mo 1.5*	*mo 3*	*mo 4.5*	*mo 6*
**TIMEPOINT**	$-t_1$	t	t_1	t_2	t_3	t_4
**ENROLLMENT:**						
**Eligibility screen**	X					
**Informed consent**	X					
**Demographic details**	X					
**Medical history**	X					
**3-day dietary records**	X					
**Allocation**		X				
**INTERVENTIONS:**						
**Vegetarian Diet**		◆———	———	———◆ ◆- - -	- - -	- - -◆
**Mediterranean Diet**		◆- - -	- - -	- - -◆ ◆———	———	———◆
**ASSESSMENTS:**						
Anthropometric measurements		X	X	X	X	X
Body composition		X	X	X	X	X
Blood samples		X		X		X
Stool samples		X		X		X
**Food Acceptability Questionnaire**				X		X
**Adherence questionnaires**			X	X	X	X
**24-hour diet recall**		◆———	———	———	———	———◆
