# Supplementary material for: Mediterranean versus vegetarian diet for cardiovascular disease prevention (the CARDIVEG study): study protocol for a randomized controlled trial
Source: Trials. 2016 May 4;17:233. doi: 10.1186/s13063-016-1353-x (PMC4855805; doi:10.1186/s13063-016-1353-x)
Supplement: Additional file 3: — Informed consent form. (DOC 220 kb) [file 13063_2016_1353_MOESM3_ESM.doc]

| Titolo studio | **Prevenzione CARdiovascolare con la DIeta VEGetariana (CARDIVEG)** | | | | | | | | |
| --- | --- | --- | --- | --- | --- | --- | --- | --- | --- |
| Versione del protocollo | …….. | | | | | | | | |
|  | | | | | | | | | |
| Io sottoscritto | | …………………………………… | | | | | ………………………….……… | | |
|  | | Cognome | | | | | nome | | |
| età (anni) | ………………… | |  |  | data di nascita | | ……. / ……. / ……. | | |
| Indirizzo | ………………………………………………………..……… | | | | | | | | ………………… |
|  | P.zza / Via / V.le | | | | | | | | Numero Civico |
| Città | …………………..………………………. | | | | | telefono | | ………………………….. | |
| Dichiaro di:   - partecipare volontariamente allo studio di cui mi sono stati spiegati e di cui ho compreso lo scopo, le procedure alle quali potrò essere esposto, i possibili rischi e i benefici e le possibili alternative - aver preso visione delle “Informazioni scritte per il paziente” facenti parte di questo consenso, che confermano quanto mi è stato detto sullo studio - aver avuto l’opportunità di porre domande chiarificatrici e di aver avuto risposte soddisfacenti - aver avuto tutto il tempo necessario prima di decidere se partecipare o meno - non aver avuto alcuna coercizione indebita nella richiesta del Consenso - acconsentire/non acconsentire a che il mio medico curante venga informato dallo sperimentatore circa la mia partecipazione allo studio sperimentale in oggetto - ai sensi del Decreto legislativo 30.6.03 n. 196 codice privacy, autorizzo il proponente di questo studio, a sottoporre a trattamento (nel senso specificato dalla legge) i dati personali e sensibili che mi riguardano, forniti allo sperimentatore, in quanto necessari alla mia partecipazione allo studio in oggetto. | | | | | | | | | |
| Data ………. / ………. / ……….  Firma del paziente (adulto, minore maturo) ....................................................  Firma di entrambi genitori in caso di soggetto minore .....................................................  .....................................................  Firma del rappresentante legale (in caso di paziente inabilitato, interdetto o con amministratore di sostegno) ..................................................... | | | | | | | | | |

|  |  |  |  |
| --- | --- | --- | --- |
| Io sottoscritto Prof./Dr. |  | ………………………………… | ………………………..… |
|  | | Cognome | Nome |
| Dichiaro che il paziente ha firmato spontaneamente la sua partecipazione allo studio  Dichiaro inoltre di:   - aver fornito al paziente esaurienti spiegazioni in merito alle finalità dello studio, alle procedure, ai possibili rischi e benefici e alle possibili alternative; - aver verificato che il paziente abbia sufficientemente compreso le informazioni fornitegli - aver lasciato al paziente il tempo necessario e la possibilità di fare domande in merito allo studio - non aver esercitato alcuna coercizione od influenza indebita nella richiesta del Consenso | | | |
| Data ………. / ………. / ……….  .........................................................................  Firma del Medico che ha informato il paziente  e richiesto il consenso informato | | | |
